# Exposure to Second-hand Smoke During Pregnancy and Preterm Delivery

**DOI:** 10.5812/ijhrba.7630

**Published:** 2013-03-12

**Authors:** Mahdiyeh Mojibyan, Mehran Karimi, Reza Bidaki, Parivash Rafiee, Asghar Zare

**Affiliations:** 1 Department of Gynecology, Shahid Sadoughi University of Medical Sciences and Health Services, Yazd, IR Iran; 2 Department of Pediatrics, Shahid Sadoughi University of Medical Sciences and Health Services, Yazd, IR Iran; 3 Department of Psychiatry, Rafsanjan University of Medical Sciences, Rafsanjan, IR Iran; 4 General practitioner, School of medicine , Shahid Sadoughi University of Medical Sciences and Health Services, Yazd, IR Iran; 5 Health Center of Yazd Province, Shahid Sadoughi University of Medical Sciences, Yazd, IR Iran

**Keywords:** Smoke, Pregnancy, Preterm Delivery, low birth weight

## Abstract

**Background:**

Prematurity is an issue related to increasing the neonatal morbidity and mortality and smoking pregnant women cause the risk of low birth weight and prematurity increase, compared to non-smoking ones.

**Objectives:**

This study investigates second-hand smoke (SHS) exposure’s effects over pregnant women on gestational age and birth weight.

**Materials and Methods:**

In this descriptive-analytic study, 205 women referred to both public and private hospitals in the third trimester were questioned about second-hand smoke (SHS) exposure during pregnancy. In addition to birth weight and gestational age, other variables including mother’s education and job and sex of the newborns were also assessed.

**Results:**

Of all 205 women, 43 (20.97%) women exposed to SHS during pregnancy and 162 (79.02%) women did not. In SHS exposure group, 11 infant (25.6%) and in non- SHS exposure group, 17 infant (10.5%), were born prematurely (< 37 weeks) (P = 0.01). Also birth weight of newborn in non-SHS exposure group was 118 gram more than other group but the differences were not significant (P = 0.09).

**Conclusions:**

Our findings showed that the secondhand smoke (SHS) exposure of pregnant women may be significantly associated with early preterm delivery.

## 1. Background

There are many identifiable causes of preterm birth such as multi parity, placental dysfunction, bicornuate uterus, preeclampsia, low socioeconomic status, maternal under nutrition, anemia, inadequate prenatal care, obstetric complications, teenage pregnancies, short interval of pregnancy, maternal size and maternal smoking (1). Cigarette smoking often associated with intrauterine growth restriction (1). The risk of spontaneous abortion for the heavy smoker is estimated to be as much as 1.7 times more than that of the non-smokers and the risk of congenital abnormality for babies born of smoking mothers is estimated to be as much as 2.3 times more than that of the nonsmokers (2). Heavy paternal smoking increases the risk of early pregnancy loss through maternal and/or paternal exposure (3). Mothers who smoke during pregnancy are highly likely to have a LBW infant, and LBW infants of smoking mothers weigh an average of 150 to 250 g less than nonsmoking mothers’ infants (4). It is shown that children of nonsmoking mothers generally perform better than the two smoking groups with regard to speech and language skills, intelligence, visual / spatial abilities and rating of mother's behavior tests. Moreover, the performance of passive smokers’ children, in most areas, found to be between the active smoking and nonsmoking groups (5). Studies showed the neurotoxic effects of prenatal tobacco exposure on newborn neurobehavioral (6). Second-hand smoke (SHS) exposure is the main cause of premature death and disease among women and children (7). In fact, SHS is exhaled smoke, the smoke from burning tobacco, filter or mouthpiece end of a cigarette, pipe or cigar (8). It also includes smoke fills restaurants, offices or other enclosed spaces when people burn tobacco products such as cigarettes and water pipes. There is no safe level of SHS exposure (9). Tobacco smoke contains many poisons, including nicotine (a pesticide), carbon monoxide, ammonia, formaldehyde, hydrogen cyanide, nitrogen oxides, phenol, sulfur dioxide, and others (8). More than 126 million nonsmokers are exposed to SHS in the United States, and home smoking comprises the most common site of SHS exposure (6, 8, 10). In the US, the proportion of women who reported smoking during pregnancy has decreased by 50% over the past 15 years (from 20% in 1989 to 10% in 2004); however, with regard to social undesirability of smoking during pregnancy, many experts question the accuracy of self-reported tobacco use in this regard (11). One of the significant consequences of prenatal tobacco exposure is sensitization of the fetal brain against nicotine, which results in increasing likelihood of addiction when the brain is exposed to nicotine at a later age (12). Population-based human studies have demonstrated the relationship between prenatal tobacco exposure and early tobacco experimentation and increasing likelihood of tobacco use in adolescents as well. (13). The toxins in SHS directly cause harmful effects on the fetus (14-17). Nicotine is known to be vasoactive and is thought to reduce fetal circulation via the placenta (18-23). Cotinine, a major metabolite of nicotine, has been measured in follicular fluid and amniotic fluid. Carbon monoxide is known to deplete fetal oxygen supplies (24-27). Second-hand smoke exposure causes 600000 premature deaths per year (9).

## 2. Objectives

The goal of our study is to examine the association of pregnant women and secondhand smoke exposure (or passive smoking), during the first and second trimester of pregnancy, with birth weight and gestational age.

## 3. Materials and Methods

In this descriptive-analytic study, 205 women between 16 and 40 old years were questioned about secondhand smoke (SHS) exposure during their pregnancy. Pregnant women, referred to Sadoghi (public) and Mojibian (Private) hospitals in the third trimester from January till July, 2009 participated in the study. Non-random simple method was used as sampling procedure. Based on statistical calculations (α = 0.05), sample size consisted of 205 people. 

As recorded in the neonate’s medical records, Low birth weight-less than 2,500 g at birth- was defined as a neonate and live born infants, delivered before 37 weeks from the 1st day of the last menstrual period (LMP), were termed premature. In addition to birth weight and gestational age assessment, other variables including mother’s education, mother’s job and sex of newborn were also evaluated. After data collection, through SPSS 16 software and using independent *t*-test, Man-Whitney and Chi-square test data analysis have shown the significant level of P < 0.05 in the present study.

## 4. Results

The mean age of women was 25.93 years (SD = 5.14 y/o); 86.9% of them reported having a high school degree and below and 13.1% possessing a bachelor degree.

Also 13 women (6.3%) were workers and others were house wives (Table 1). The mean birth weight of the neonates was 3132 g (SD = ± 404 g). Furthermore, 47.8% of the participants had baby boys (n = 98); 32.8% had vaginal deliveries (n = 67); 67.2% had caesarean sections (n = 137); and 6.4% had LBW neonates (n = 13). In pregnant women with and without SHS exposure, 21 (48.8%) and 77 (47.5%) of offspring were male respectively (P = 0.879). 43 women were SHS exposure during pregnancy and 162 were not. In SHS exposure group, 11 infants (25.6%) and in non-SHS exposure group, 17 (10.5%) infants were born prematurely. (< 37 weeks) (P = 0.01). Mean Birth weight in the infants of mothers who had SHS exposure were 3038 ± 491 g and in non-SHS exposure were (3156 ± 375 g, P value = 0.09) (Table 2).

**Table 1.** Demographic Characteristics of Pregnant Women and Their Newborn

	Number (%)
**Sex of newborn**	
Boy	98 (47.8)
Girl	107 (51.9)
**Mother’s education**	
High school degree and below	179 (86.9)
Bachelor degree	27 (13.1)
**Mother’s Job**	
House wife	193 (93.7)
Worker	13 (6.3)

**Table 2.** Gestational Age Distribution in Pregnant Women With and Without SHS Exposure

	SHS Exposure		Total
	**Yes. Count (%)**	**No. Count (%)**	
**Gestational age**			
under 38	11 (25.6)	17 (10.5)	28 (13.7)
38-40	32 (74.4)	143 (88.3)	175 (85.4)
Upper 40	0 (0)	2 (1.2)	2 (1)
**Total**	43 (100)	162 (100)	205 (100)

## 5. Discussion

The results of our study showed an adverse effect of SHS exposure on gestational age and birth weight at the time of delivery. This is consistent with the results of previous studies, in which the researchers found that maternal exposure to SHS was associated with prematurity and low birth weight (21, 24, 28-32). In Iran, 27.3% of men and 3.4% of women smoke cigarette. 29.3% of Iranian pregnant women are SHS exposure and 0.7% smoke cigarette, which is statistically less than US pregnant women (10%) (33). Although results of many studies significant relationship between smoking during pregnancy with preterm delivery and low birth weight but results about SHS exposure is controversial (28, 34, 35).

### 5.1. Birth weight

In our study the mean birth weight of newborn in non-SHS exposure group was 118 g more than SHS exposure group (P = 0.09). This finding is similar to some studies which found that maternal exposure to SHS was not associated with low birth weight (19, 36, 37). However, there are various findings with regard to SHS exposure and mean birth weight, some studies reported significant decrements in birth weight (9, 38, 39). Leonardi et al. reviewed 58 studies and concluded that environmental tobacco smoke (ETS) exposure was associated with a 33 to 40 g reduction in mean birth weight (29). Hanke et al. found statistically significant negative relationship between the fetal bi-parietal diameter (BPD) and serum cotinine concentration. They found that serum cotinine levels at 20-24 weeks of gestation were inversely associated with infant birth weight. With regard to serum cotinine levels below 10 ng/mL, a borderline association (P = 0.09), with infant birth weight was found (33). Also Rebagliato et al. showed that the mean birth weight of infants of women with cotinine levels > 1.7 ng/mL was 87.3 g less than that of infants of women with cotinine levels in the range 0.0-0.5 ng/mL (P = 0.048) (31). Shekibazadeh showed no significant correlation between pregnancy SHS exposure and head circumference and length of newborn; however birth weight was significantly lower in SHS exposure group (38).

### 5.2. Prematurity and Small for gestational age

As shown in Table 2 in our study, prematurity was significantly higher in SHS exposure group. Other studies of ETS exposure and Prematurity or small for gestational age have found varying results, from no effect to significant negative association. Results of many studies showed that ETS exposure during pregnancy is associated with an increased risk of term SGA (Small for Gestational Age) and prematurity (34, 36, 40-42). However; the results of a few studies showed that ETS exposure during pregnancy is not associated with an increased risk of term SGA (29, 43, 44). This is due to difficulties in precisely assessing exposure or different methodology in these studies. Dejin-Karlsson et al. showed that women exposed to passive smoking at home or in the workplace face the risk of delivering a small-for-gestational-age infant in significantly compared to non-exposure women. Also they concluded that passive smoking was not significantly related to low birth weight ( < 2500 g) or preterm delivery ( < 37 gestational weeks) (45). Readers should consider several limitations of our study when interpreting the results. First, we did not measure the exposure rate, patterns of exposure (husband smoking or smoking by other members of family). Second, we also did not determine whether the environment of tobacco smoke exposure change during pregnancy. Third, we did not measure a biomarker of tobacco smoke exposure.

Although we didn’t study SHS exposure rate, there are sufficient evidences from many studies in this regard. There is a significant negative association with exposure rate and gestational age and birth weight (24, 29, 32, 34, 42). We found only one study that showed no significant association between increasing daily exposure to ETS and reducing the birth weight (46). In our study, all exposure to ETS measured only at home and even some studies showed no difference between indoor and outdoor ETS exposure effects on SGA and/or birth Weight (32, 47). Although, Fortier showed that single passive exposure to tobacco smoke at home was not related to SGA. However, small increments in risks were observed in only passive smoking women at work, and the risks increased consistently with weekly duration, number of weeks, and intensity of exposure (48). Our findings showed that the SHS exposure of pregnant women is significantly associated with preterm delivery. Fortunately there are many antismoking rules imposed over public places, offices, and mosques in Iran. Moreover, there are confining laws prohibiting cigarette ads in newspapers and media and people usually protest indoor smokers. In our province all health houses and centers are completely developed. So we suggest using this system for prenatal screening of pregnant SHS exposure.
